# One-Pot Synthesis of Melamine Formaldehyde Resin-Derived *N*-Doped Porous Carbon for CO_2_ Capture Application

**DOI:** 10.3390/molecules28041772

**Published:** 2023-02-13

**Authors:** Qiyun Yu, Jiali Bai, Jiamei Huang, Muslum Demir, Ahmed A. Farghaly, Parya Aghamohammadi, Xin Hu, Linlin Wang

**Affiliations:** 1 Key Laboratory of the Ministry of Education for Advanced Catalysis Materials, Zhejiang Normal University, Jinhua 321004, China; 2 Department of Chemical Engineering, Osmaniye Korkut Ata University, Osmaniye 80000, Turkey; 3 TUBITAK Marmara Research Center, Material Institute, Gebze 41470, Turkey; 4 Chemical Sciences and Engineering Division, Argonne National Laboratory, Lemont, IL 60439, USA; 5 Pritzker School of Molecular Engineering, The University of Chicago, Chicago, IL 60637, USA; 6 Chemistry Department, Faculty of Science, Assiut University, Assiut 71516, Egypt; 7 Key Laboratory of Urban Rail Transit Intelligent Operation and Maintenance Technology and Equipment of Zhejiang Province, College of Engineering, Zhejiang Normal University, Jinhua 321004, China

**Keywords:** N-rich porous carbons, CO_2_ adsorption, KOH activation, single-step reaction

## Abstract

The design and synthesis of porous carbons for CO_2_ adsorption have attracted tremendous interest owing to the ever-soaring concerns regarding climate change and global warming. Herein, for the first time, nitrogen-rich porous carbon was prepared with chemical activation (KOH) of commercial melamine formaldehyde resin (MF) in a single step. It has been shown that the porosity parameters of the as-prepared carbons were successfully tuned by controlling the activating temperature and adjusting the amount of KOH. Thus, as-prepared N-rich porous carbon shows a large surface area of 1658 m^2^/g and a high N content of 16.07 wt%. Benefiting from the unique physical and textural features, the optimal sample depicted a CO_2_ uptake of up to 4.95 and 3.30 mmol/g at 0 and 25 °C under 1 bar of pressure. More importantly, as-prepared adsorbents show great CO_2_ selectivity over N_2_ and outstanding recyclability, which was prominently important for CO_2_ capture from the flue gases in practical application. An in-depth analysis illustrated that the synergetic effect of textural properties and surface nitrogen decoration mainly determined the CO_2_ capture performance. However, the textural properties of carbons play a more important role than surface functionalities in deciding CO_2_ uptake. In view of cost-effective synthesis, outstanding textural activity, and the high adsorption capacity together with good selectivity, this advanced approach becomes valid and convenient in fabricating a unique highly efficient *N*-rich carbon adsorbent for CO_2_ uptake and separation from flue gases.

## 1. Introduction

As of September 2022, the atmospheric carbon dioxide was recorded at 414.57 parts per million [1]. This colossal amount of CO_2_ has become a major threat to Earth’s environment and sustainable development. To combat this, many countries including the United States have set a goal to reduce greenhouse gas pollution by 50% from 2005 levels by 2030, and eventually achieve net zero emissions by 2050 [2]. With energy consumption and demands continuing to increase, the mitigation of carbon dioxide emissions has become a critical necessity in order to achieve these goals. Current strategies for large-scale mitigation are the capture of CO_2_ from the atmosphere through pressure or vacuum swing adsorption (P/VSA) processes [3], precombustion capture, post-combustion capture via amine solutions, and oxyfuel combustion [4,5]. Post-combustion capture uses amine absorption technology and involves the aqueous scrubbing of flue gas streams. These scrubbing systems absorb CO_2_ from flue gas, strip and release the CO_2_ in concentrated form, and allow the recovery of the original amine solvent [6]. All of these techniques represent great technological advances for CO_2_ capture. However, these methods come with drawbacks. For example, amine absorption requires a large amount of heat in order to regenerate the solvent, reducing the overall efficiency and increasing the capital cost of the system [7]. Due to the excessive cost, complicated set-up, and high energy consumption of the current methods, more mitigation strategies need to be explored. Gas–solid adsorption is a promising alternative due to its lower energy demands and less extreme operating conditions [8,9,10,11,12].

Over the past few decades, many high-surface-area materials such as metal–organic frameworks (MOFs) [13,14], zeolites [15], porous polymers [16,17,18], amine-modified materials [19,20], covalent–organic frameworks (COFs) [21], and different forms of porous carbons [22,23,24,25,26,27,28,29,30,31] have been explored as possible CO_2_ adsorbents. Among these, porous carbon adsorbents are considered to be the most promising based on their superb characteristics such as regulated porous structure, abundant precursors, robustness, high surface area, high hydrophobicity, and energy-efficient recovery where much less energy is required for regeneration compared to conventional capture techniques [32,33].

Two techniques have been explored to improve the selectivity and adsorption capacity of these carbonaceous materials. One of these techniques is to improve the pore structure of the material. It has been reported that microporous carbons with high surface areas have higher CO_2_ adsorption capacity [34]. Previous studies also confirm that the use of heteroatom dopants such as nitrogen, sulfur, and oxygen on the surfaces of porous carbon materials can improve their ability to capture CO_2_ [35,36,37]. Heteroatoms can improve the electrical properties of a porous material and increase carbon’s overall affinity for CO_2_ compared to non-doped carbons. When a carbon-based sample is functionalized with nitrogen, it favors the adsorption of carbon dioxide through acid–base interactions, quadrupolar interactions, and hydrogen-bond formation [38,39,40].

Multiple techniques have been explored in order to dope carbon materials with heteroatoms. These methods can be identified as either post-treatment or in situ doping. With post-treatment techniques, the carbon-based materials are treated with nitrogen-containing compounds such as urea [41], ammonia [42], and melamine [43] at high temperatures. This step incorporates nitrogen functional groups into the material, and it can be used to produce activated or porous carbon samples. With in situ methods, nitrogen-containing compounds are used as precursors to directly produce nitrogen-doped carbonaceous materials [44,45]. Successful functionalization of nitrogen to carbon samples can significantly enhance their abilities to adsorb and capture CO_2_. To the best of our knowledge, there is no direct utilization of commercial melamine formaldehyde resin (MF) via chemical activating agents for the application of CO_2_ capture. Thus, the present study fills this gap and this is the main novelty of the present research.

Herein, we, for the first time, prepared nitrogen-rich porous carbon adsorbents using a commercial melamine formaldehyde resin (MF) and KOH as the precursor and activating substance, respectively. The textural activity and nitrogen content of the carbons were regulated by adjusting the amount of the KOH and the activating temperature, which eventually affected the CO_2_ adsorption capacity and selectivity of CO_2_ over N_2_. Additionally, we fully investigated the relation between CO_2_ adsorption and porous properties for the as-prepared carbon-based adsorbents. Other parameters such as CO_2_/N_2_ selectivity, the heat of adsorption, CO_2_ adsorption kinetics behavior, stability, and dynamic CO_2_ capture performance were also examined.

## 2. Results and Discussion

### 2.1. Morphological, Phase Structural, and Surface Chemical Properties

**Figure 1:**
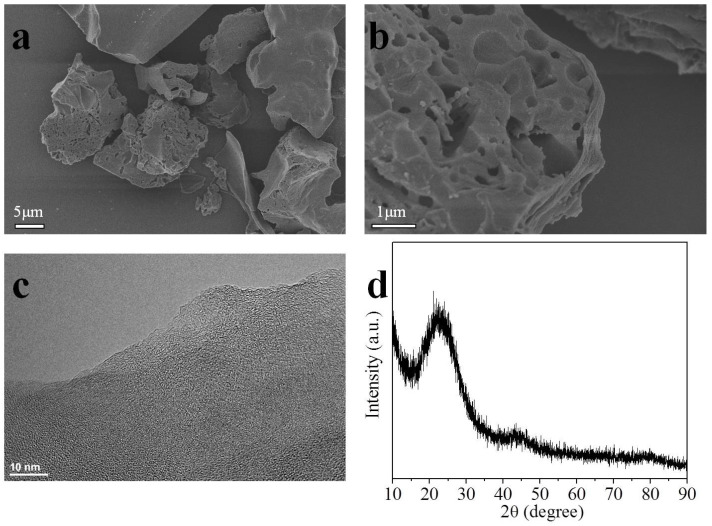
SEM images (**a**,**b**); TEM image (**c**); and XRD pattern (**d**) of MFC-700-0.2.

Scanning electron microscopy (SEM) images were used to perceive the morphology and microscopic structures of carbons. The surface morphology of the representative MFC-700-0.2 sample is presented at different magnifications in Figure 1a,b. Notably, the surface of the MFC-700-0.2 sample looks rough and an obvious porous structure (eroded cavities) is prominent, which is critically beneficial for holding a vast amount of CO_2_ gas. The transmission electron microscopy (TEM) image of the same sample is shown in Figure 1c, which displays a nanoscale rich honeycomb structure. X-ray diffraction (XRD) was applied to assess the crystal framework of the selected MFC-700-0.2 porous carbon, as depicted in Figure 1d. The XRD spectra of the MFC-700-0.2 present one intense and one broad peak placed at 2θ values of 23 and 43°, which were assigned to (002) and (100) reflections of graphitic structure [46].

**Figure 2:**
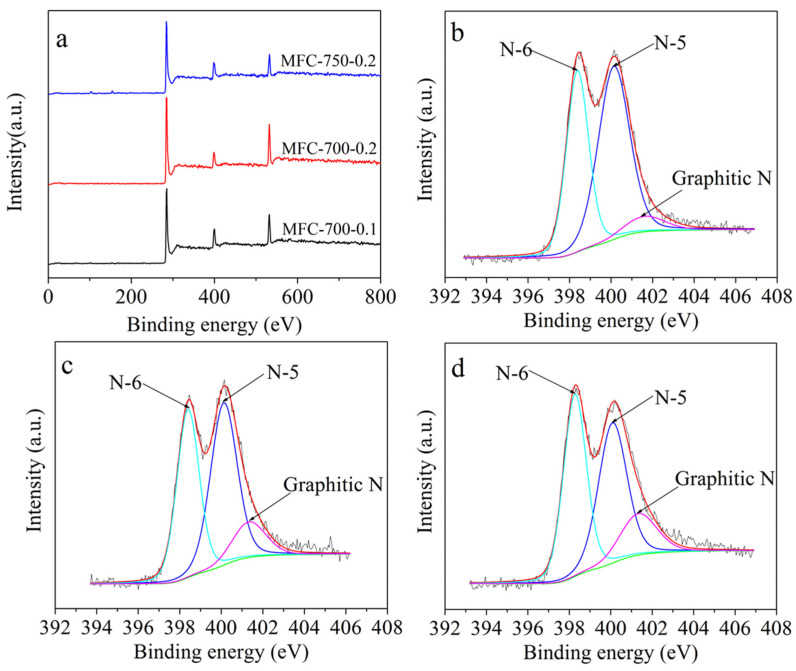
XPS survey (**a**) of selected adsorbents; XPS N 1s of (**b**) MFC-700-0.1, (**c**) MFC-700-0.2, and (**d**) MFC-750-0.2. In (**b**,**c**), the black line is the original data and the red line is the fitting curve, the green line is the background.

The elemental content of C, N, and H of the MF-derived porous carbon samples was obtained by elemental analysis (Table 1). It was noticed that up to 16.07 wt% N was held in the as-prepared carbon adsorbents when annealed between 600 and 750 °C, which is an outstanding amount of nitrogen functionality compared to similar materials. X-ray photoelectron spectroscopy (XPS) was conducted to assess the chemical environment in as-prepared adsorbents. As indicated in Figure 2a, the survey scan spectra of the MFC-700-0.1, MFC-700-0.2, and MFC-750-0.2 samples presented peaks located at around 284.5, 398.3, and 531.2 eV, signifying the main presence of C, N, and O atoms. The C1s spectrum can be deconvoluted into signals for C–C (284.5 eV), C–N (285.2 eV), C–O (286.2 eV), and C=O (288.6 eV) [47] (Figure S1, Supplementary Materials). The N 1s core-level XPS spectra of the MFC-700-0.1, MFC-700-0.2, and MFC-750-0.2 samples depicted three prominent peaks placed at 398.4, 400.3, and 401.4 eV, which refer to pyridinic (N-6), pyrrolic (N-5), and graphitic N, respectively [48,49,50]. The fully N functional group contents for these adsorbents are provided in Table S1 (Supplementary Materials). Previous studies reported that pyridinic and pyrrolic nitrogen functional groups integrated into a carbon framework led to strong Lewis basicity, in other words, enhanced electron donor activities, which are favorable to the CO_2_ adsorption capacity [51,52,53].

### 2.2. Porous Textual Properties

**Figure 3:**
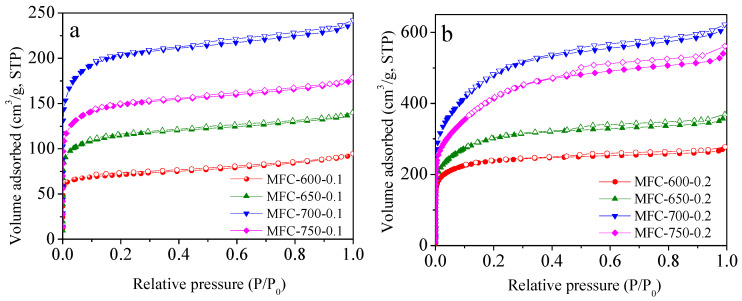
N_2_ sorption isotherms of the samples prepared at KOH/MF ratio of (**a**) 0.1 and (**b**) 0.2. Filled and empty symbols represent adsorption and desorption branches, respectively. STP: standard temperature and pressure.

The textural properties of the MFC-T-m samples were analyzed by N_2_ sorption, and isotherms are presented in Figure 3, with complete parameters provided in Table 1. The semi-logarithmic N_2_ sorption isotherms of all the samples are included in Figure S2. It is shown in Figure 3 that the adsorption branches of all samples in N_2_ isotherms are mostly overlapped with desorption branches, and sorption trends tend to be a horizontal plateau at a very low pressure of 0.1, signifying a typical type I isotherm with a high amount of micropore formation in the carbon framework. Furthermore, in the case of the MFC-650-0.2, MFC-700-0.2, and MFC-750-0.2 samples, isotherms depict a wide knee with adsorbed amounts significantly rising as the P/P_0_ value reaches 0.3, indicated the existence of small-sized mesopores and certain large-sized micropores [54]. Additionally, these samples slightly present typical type IV isotherms with a small hysteresis loop at a pressure of 0.9–1.0, indicating the presence of some macropore structure. [55,56].

**Figure 4:**
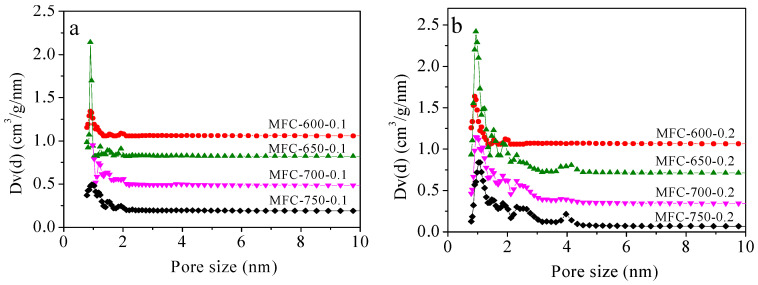
Pore size distribution of the samples prepared at KOH/MF ratio of (**a**) 0.1 and (**b**) 0.2. Dv(d) = dV/dD.

The pore size distributions (PSDs) of the MFC-T-m samples are shown in Figure 4. As observed, almost all porous carbons depict highly concentrated micropores in the range of 0.4 to 0.6 nm. On the other hand, the MFC-650-0.2, MFC-700-0.2, and MFC-750-0.2 samples present a mesopore distribution in the 2.2 to 4.0 nm range. Thus, the carbons have a hierarchical pore structure, i.e., abundant micropore structure together with a certain amount of meso- and macroporosity. Different porous characteristics such as the specific BET surface area (S_BET_), total pore volume (V_0_), and micropore volume (V_t_) values of the adsorbents are provided in Table 1. It has been found that as the activating temperature increases from the 600 to 700 °C, these porous parameters significantly rise; however, upon further increasing the activating temperature to 750 °C, these values slightly decrease, most probably owing to the destructive effect of the chemical activating agent (KOH) at elevated temperatures, which results in destruction of existing pores and then leads to a shrinkage of microporosity. This is consistent with previous literature studies [57,58]. Furthermore, we investigated the effect of the amount of activating agents on the pore formation and concluded that as the KOH/MF mass ratio increased from 0.1 to 0.2, the textural properties of the carbons notably enhanced thanks to a certain amount of KOH reaching the large-area boundary and opening more and more porosity within the carbon structure.

Since the narrow micropores (<1 nm) were well-recognized as the key factor that determines the CO_2_ uptake of porous carbons under 1 bar and 25 °C conditions [59,60], herein, the volume of narrow micropores (V_n_) in the MFC-T-m samples was calculated using the Dubinin−Radushkevich (D−R) equation based on their CO_2_ adsorption data at 0 °C. As shown in Table 1, the changing trend of V_n_ with respect to activation temperature and KOH/MF mass ratio is the same as with the other porous parameters, with a maximum V_n_ of 0.62 cm^3^/g for MFC-700-0.2.

### 2.3. CO_2_ Adsorption Performance of the N-Doped Porous Carbons

**Figure 5:**
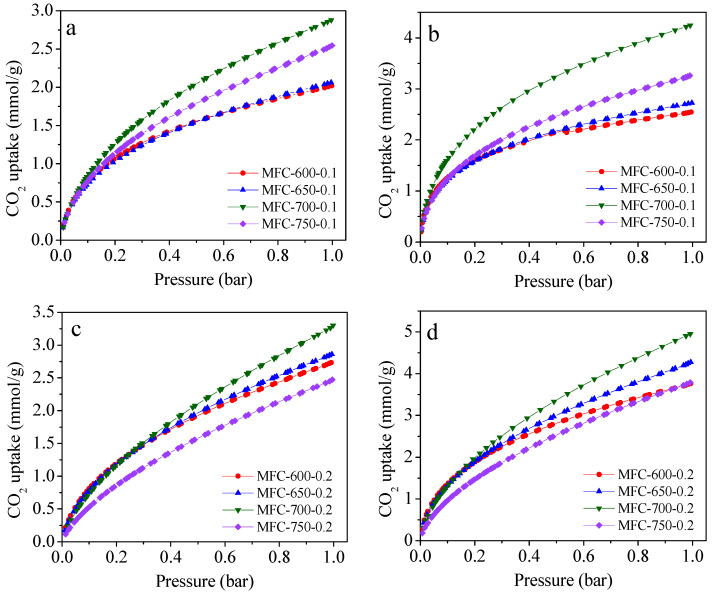
CO_2_ adsorption isotherms at 25 °C (**a**,**c**) and 0 °C (**b**,**d**) for melamine formaldehyde resin-derived N-doped carbons prepared under different conditions.

Having confirmed the existence of high nitrogen content along with advanced textural properties, the CO_2_ adsorption performances of N-rich porous carbon were investigated in depth. The CO_2_ adsorption isotherms of MFC-T-m samples are depicted in Figure 5 and corresponding CO_2_ uptake capacities are given in Table 1. It is obvious that the CO_2_ adsorption isotherms tend to continue increasing even above 1 bar, signifying that higher adsorption capacities can be achieved at elevated adsorption pressure. Another critical conclusion of the CO_2_ adsorption isotherm plot is that the adsorption capacity of all N-doped porous carbons decreased as the adsorption temperature increased from 0 to 25 °C, indicating that the adsorption mechanism predominantly occurred via an exothermic physical process [61]. To define the optimal CO_2_ adsorbent, different experimental conditions were investigated in terms of the activation temperature and the KOH/MF mass ratio. The results of the optimization parameters are as follows: Firstly, as the activating temperature rose from 600 to 650 °C, the CO_2_ capture capacity was significantly increased at both 0 and 25 °C. Further increasing the activation temperature to 700 °C, the CO_2_ capture capacity was increased again. However, a further increase in the activating temperature from 700 to 750 °C resulted in a notable decrease in the CO_2_ capture capacity due to the low textural properties since the potential porosity collapse occurred at the elevated activating temperature. We further investigated how the KOH/MF mass ratio affects CO_2_ capture performance. It was found that a mass ratio of 0.2 is the optimal amount for CO_2_ capture activity. In short, 700 °C and 0.2 were found to be optimal activating temperature and KOH/MF mass ratio. It should be pointed out that MFC-700-0.2, the optimal sample in this study, has an CO_2_ adsorption performance comparable to many classic sorbents such as porous carbons [62,63], MOFs [13], COFs [64], porous aromatic frameworks (PAFs) [65], and porous polymers [16].

**Figure 6:**
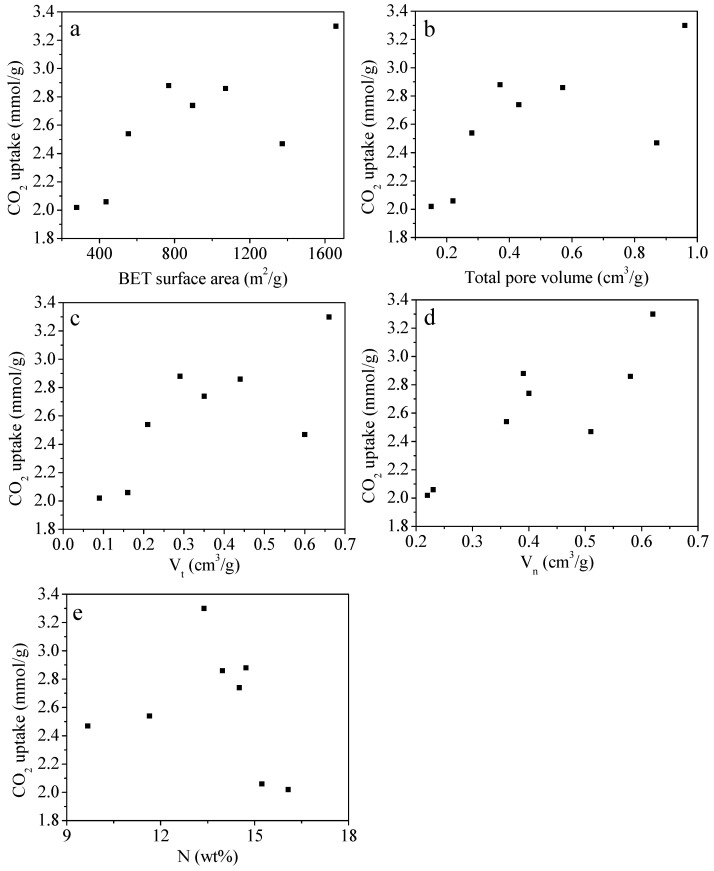
Plot of each porous properties characteristic: (**a**) S_BET_, (**b**) V_0_, (**c**) V_t_, (**d**) V_n_, and (**e**) nitrogen content versus CO_2_ uptake.

The adsorption performances of the MFC-T-m samples were further examined to understand the factors affecting CO_2_ capture activity. The correlations between S_BET_, V_0_, V_t_, V_n_, and nitrogen content versus CO_2_ uptake are depicted in Figure 6. Notably, certain correlations between CO_2_ adsorption capacity and S_BET_, V_0_, V_t_, V_n_, and nitrogen content were observed. For instance, there is a good linear relationship between the CO_2_ capacity and S_BET_, V_0_, V_t_, and V_n_. This claims that as the textural properties enhance, the CO_2_ capture performance of the adsorbents is significantly increased. This is owing to the fact that the narrow micropore feature plays a paramount role in the CO_2_ adsorption process. Moreover, there is no apparent linear correlation between N content and CO_2_ uptake capacity; however, this does not reflect the fact that the nitrogen functionality is a non-defining parameter for carbon capture applications. When we compared the CO_2_ uptake among MFC-600-0.2, MFC-700-0.1, and MFC-750-0.2, it was found that MFC-750-0.2 had the lowest CO_2_ uptake of these three samples, even though it has the most advanced porous textural properties. The higher CO_2_ uptake in MFC-600-0.2 and MFC-700-0.1 can be ascribed to their higher N content compared to MFC-750-0.2. To sum up, both the textural properties and nitrogen functionalities are responsible for the CO_2_ capture capacity of the as-prepared adsorbents. Considering the fact that this series of samples has a high nitrogen content (up to 16 wt%), but their CO_2_ adsorption capacity is lower than 4 mmol/g at 25 °C and 1 bar, we think that between narrow porosity and N content, the narrow porosity is the more important factor that determines CO_2_ uptake of carbonaceous sorbents.

**Figure 7:**
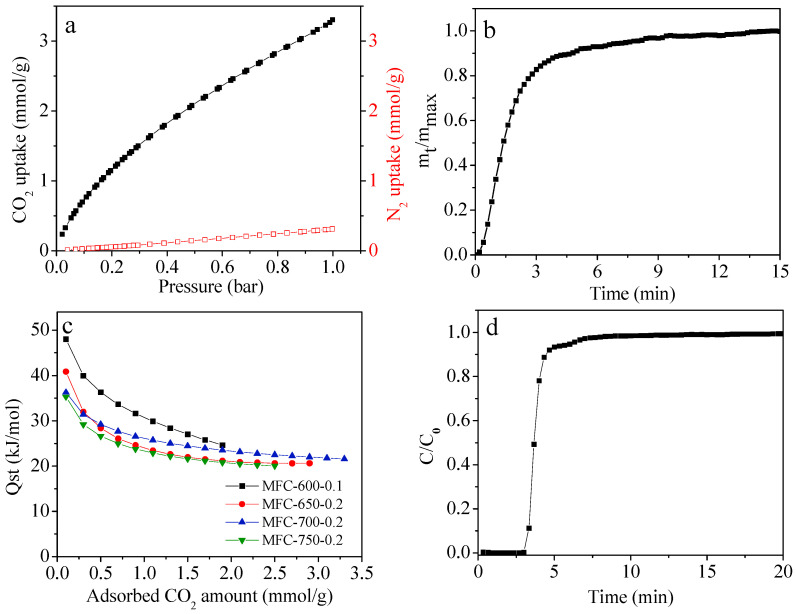
(**a**) CO_2_ and N_2_ adsorption isotherms of MFC-700-0.2 at 25 °C and 1 bar, the black line is CO_2_ isotherm and the red line is N_2_ isotherm (**b**) CO_2_ adsorption kinetics at 25 °C for MFC-700-0.2, (**c**) Qst (isosteric heats of adsorption) on selected sorbents and (**d**) breakthrough curves of MFC-700-0.2. Adsorption conditions: gas pressure = 1 bar, adsorption temperature = 25 °C, gas flow rate = 10 mL/min, inlet CO_2_ concentration = 10 vol.%.

To assess the CO_2_ separation activity of the representative MFC-700-0.2 sample, the CO_2_ and N_2_ isotherms at 25 °C are presented in Figure 7a. Notably, the adsorption capacity of CO_2_ is much higher than N_2_ gas, signifying a better selectivity of the MFC-700-0.2 sample for CO_2_ than for N_2_. The ideal adsorbed solution theory (IAST) technique [66] was used to quantitatively assess the selectivity for CO_2_ over N_2_ and the IAST selectivity was calculated as 17, which is assigned to the strengthened attraction of CO_2_ molecules on N-doped carbon.

To evaluate the kinetic activity of the optimal MFC-700-0.2 sample, the CO_2_ adsorption amount versus the adsorption time is depicted in Figure 7b and it was found that within 4.5 min, 90% adsorption saturation is obtained, demonstrating fast adsorption kinetics.

To depict the combination interaction strength between the N-doped carbon adsorbents and the CO_2_ molecules, the isosteric heats of adsorption (Qst) of selected adsorbents were calculated by the Clausius–Clapeyron equation based on the CO_2_ sorption isotherm at 0 and 25 °C. The results show that the Qst steadily declines at a relatively low CO_2_ capture, and then reaches a near plateau as the constant occupation of adsorption active sites increases with the rising CO_2_ capture, indicating the heterogeneity of interactions between the surfaces of adsorbents and CO_2_ molecules, which is possibly owing to the heterogeneity of the surface chemistry [67]. The initial Qst values were found to range from 35–48 kJ/mol, which is higher than that of pure carbon-derived adsorbents (around 18 kJ/mol), signifying the strong interaction between the CO_2_ and N-doped porous carbons. It should be noted that even though the initial Qst for these N-doped carbons is higher than 40 kJ/mol, the CO_2_ adsorption on these MFC-T-m carbons is still a physisorption process.

To further confirm the practical usability of the as-prepared adsorbent, a breakthrough experiment was conducted for the MFC-700-0.2 sample and it was found that the CO_2_ dynamic capture capacity was calculated as 0.70 mmol/g at a gas pressure of 1 bar and temperature of 25 °C along with a gas flow rate of 10 mL/min (CO_2_ concentration: 10 vol.%). It needs to be acknowledged here that a certain amount of water vapor is contained in flue gas, which could deteriorate the CO_2_ capture capacity of these N-doped carbons due to the competitive adsorption between CO_2_ and H_2_O.

**Figure 8:**
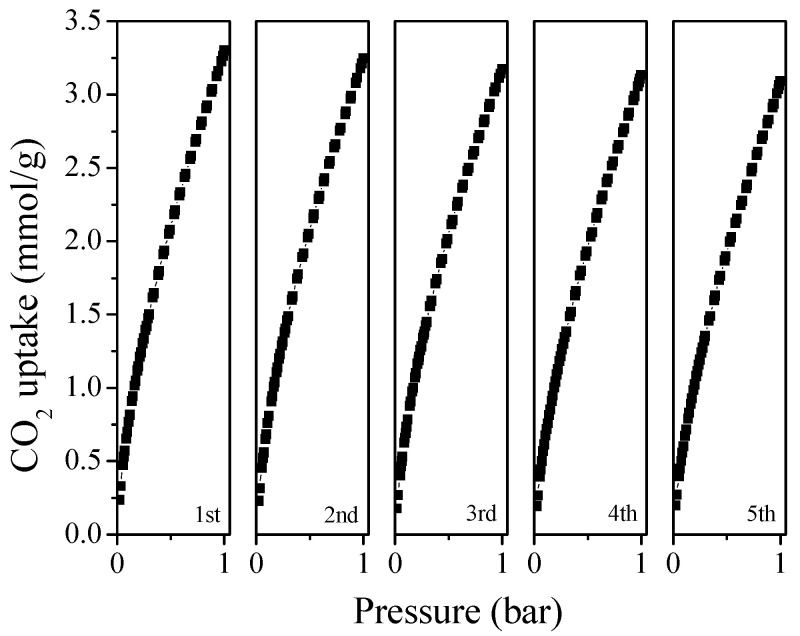
Cyclic study of CO_2_ adsorption for MFC-700-0.2.

In addition to the ideal separation activity, the recyclability performance of adsorbents is another key metric for practical CO_2_ capture. The regeneration analysis of the selected MFC-700-0.2 sample was conducted for five repeated cycles at 25 °C and 1 bar. Before each test, the sample was heated at 200 °C for 6 h in a vacuum. As depicted in Figure 8, without noticeable loss, 94% of the original CO_2_ uptake is maintained, demonstrating the excellent recyclability of as-prepared adsorbent along with the low energy requirement for the regeneration process.

## 3. Synthesis and Characterization

Commercial melamine formaldehyde resin (MF) was selected as the starting material and N-rich porous carbons were produced through a single-step reaction using direct KOH activation. Four activation temperatures (600, 650, 700, and 750 °C) and two KOH/MF ratios (0.1 and 0.2) were used for the optimization process. The resulting sorbents were labeled as MFC-T-m, where T represents the activation temperature and m represents the KOH/MF ratio. The yield of the carbon materials varied from 15.96 to 2.07 wt%.

### 3.1. KOH Activation

In a typical preparation, 2 g melamine formaldehyde resin (MF) was combined with a solution that contained 0.4 g KOH. After stirring vigorously for 8 h, the mixture was left overnight to dry at 120 °C in an oven. Afterwards, the sample was heated to 200 °C and then held at this temperature for 1 h, then the temperature was elevated to 700 °C and maintained at this temperature for 2 h. The activation was performed at a heating rate of 5 °C/min and a nitrogen flow rate of 400 mL/min. After activation, the sorbent was washed with distilled water until the pH of the filtrate was close to 7. The wet sample was dried under vacuum at 150 °C for 24 h. The final product was named MFC-700-0.2.

### 3.2. Characterization

Powdered X-ray diffraction (XRD) patterns were carried out on a PHILIPS PW3040/60 powder diffractometer (PHILIPS, Amsterdam, Netherland) using CuKα radiation (λ = 0.15406 nm). Scanning electron microscopy (SEM Hitachi S-4800, Hitachi, Tokyo, Japan) was used to observe the morphology of the samples of carbon materials. Further details of the pore structure were determined by transmission electron microscopy (TEM, JEOL-2100F, JEOL, Kyoto, Japan) operated at 200 kV. The CHN elements were analyzed using a VarioEL III Elemental Analyzer (Elementar, Hanau, Germany). Nitrogen adsorption and desorption isotherms were measured on a 3H-2000PS2 sorption analyzer (Beishide, Beijing, China) at −196 °C. Ultrahigh-purity N_2_ (99.999%, Shanghai Pujiang Gas Co., Ltd., Shanghai, China) was used for measurements. The samples were degassed under vacuum at 200 °C for at least 12 h prior to measurement. The specific surface area (S_BET_) was determined using the multipoint Brunauer–Emmett–Teller (BET) method based on the adsorption data in the relative pressure range between 0.001 and 0.09. The total micropore volume (V_t_) was derived from the N_2_ adsorption data using the t-plot method, and the total pore volume (V_0_) was estimated from the amount of liquid nitrogen adsorbed at a relative pressure of 0.99. The pore size distribution was calculated through the density functional theory (DFT) method (slit pore, equilibrium model). Additionally, X-ray photoelectron (XPS) measurements were conducted using an AXIS Nova spectrometer from Kratos Inc. (Kratos, Manchester, UK) equipped with a monochromatic Al Kα X-ray source (1486.6 eV). The XPS survey spectra were obtained with a pass energy of 160 eV, and high-resolution spectra were recorded with a pass energy of 40 eV. The XPS sub-peaks were deconvolved using a non-linear least squares curve-fitting program (Peak-Fit version 4) that utilized a Gaussian–Lorentzian mix function and Shirley background subtraction.

The CO_2_ adsorption isotherms were measured using a Beshide 3H-2000PS2 sorption analyzer at 0 °C and 25 °C. Pure CO_2_ (99.99%, Shanghai Pujiang Gas Co., Ltd., Shanghai, China) was used for adsorption. Prior to each adsorption experiment, the sample was degassed for 12 h at 200 °C to remove the guest molecules from the pores. The volume of narrow micropores (with sizes < 1 nm), Vn, was calculated from CO_2_ adsorption at 0 °C using the Dubinin–Radushkevich (D-R) equation. The measurements were repeated for each sample, until the values fell within ±2% of each other.

### 3.3. Measurement of Dynamic CO_2_ Uptake of the Sorbents

The dynamic CO_2_ uptake of the sorbents was evaluated in a fixed-bed reactor, as shown in Scheme S1, at 1 bar and 25 °C. The sample was first heated to 100 °C under N_2_ flow (20 mL/min) for 1 h. After the temperature was reduced to 25 °C, the gas flow was switched from nitrogen to a 10% CO_2_/N_2_ mixture (10 mL/min). The effluent gases were monitored in real time using an Agilent 7820A gas chromatograph equipped with a thermal conductivity detector (TCD). The dynamic CO_2_ capture capacity of each sorbent was calculated from the breakthrough curves.

### 3.4. Measurement of CO_2_ Adsorption Kinetics

The CO_2_ adsorption kinetics was measured using a NETZSCH STA 449C thermogravimetric analyzer (NETZSCH, Selb, Germany). The sample (approximately 5 mg) was purged with a He stream at 200 °C for 1 h, then cooled to the experimental temperature of 25 °C. CO_2_ gas was then introduced into the test chamber at a flow rate of 50 mL/min, and the change in weight over time was saved.

## 4. Conclusions

In this work, N-rich porous carbon with good porosity was successfully fabricated by a one-step chemical activation method using commercial melamine formaldehyde resin (MF) as both a carbon and nitrogen source. As-prepared N-doped porous carbons present great textural properties and enhanced nitrogen functionality on their surfaces. As expected, the resultant N-doped carbon adsorbent exhibit a CO_2_ uptake of up to 4.95 and 3.30 mmol/g at 0 and 25 °C (under 1 bar of pressure), respectively, which are equal to or even higher than those of similar reported CO_2_ adsorbents. The full CO_2_ capture analysis results show that a combination of textural activity and functional surface chemistry predominantly determines the carbon capture performance. However, the textural property of carbons is a more important factor than surface functionalities in determining CO_2_ uptake under ambient conditions. Furthermore, the N-doped carbon adsorbent exhibited excellent CO_2_ selectivity, moderate isosteric heat of adsorption, and satisfactory recyclability. Considering these confident outcomes such as the low-cost preparation as well as the aspect of environmental friendliness, as-prepared N-rich porous carbons are believed to be promising adsorbents for separating CO_2_ from flue gas. Regarding future direction, the practical application of the present material will be conducted.

## Figures and Tables

**Table 1:** Porous textural, elemental compositions, and CO_2_ uptakes of adsorbents derived from melamine formaldehyde resin under different conditions.

Sample	S_BET_^a^ (m^2^/g)	V_0_^b^ (cm^3^/g)	V_t_^c^ (cm^3^/g)	V_n_^d^ (cm^3^/g)	N (wt%)	C (wt%)	H (wt%)	CO_2_ Uptake (mmol/g)	
								25 °C	0 °C
MFC-600-0.1	278	0.15	0.09	0.22	16.07	58.18	3.86	2.02	2.55
MFC-600-0.2	895	0.43	0.35	0.40	14.51	57.94	3.59	2.74	3.76
MFC-650-0.1	435	0.22	0.16	0.23	15.23	59.64	3.12	2.06	2.72
MFC-650-0.2	1070	0.57	0.44	0.58	13.97	59.11	3.82	2.86	4.28
MFC-700-0.1	768	0.37	0.29	0.39	14.72	60.65	3.41	2.88	4.25
MFC-700-0.2	1658	0.96	0.66	0.62	13.38	64.13	4.04	3.30	4.95
MFC-750-0.1	554	0.28	0.21	0.36	11.64	58.26	2.60	2.54	3.26
MFC-750-0.2	1373	0.87	0.60	0.51	9.67	60.34	2.98	2.47	3.79

^a^ Surface area was calculated using the BET method at P/P_0_ = 0.005–0.05. ^b^ Total pore volume at P/P_0_ = 0.99. ^c^ Evaluated by the t-plot method. ^d^ Pore volume of narrow micropores (<1 nm) obtained from the CO_2_ adsorption data at 0 °C.

## Data Availability

The data presented in this study are available on request from the corresponding author.

## Supplementary Materials

The following supporting information can be downloaded at: https://www.mdpi.com/article/10.3390/molecules28041772/s1, Scheme S1. Schematic of the fixed-bed reactor system. Figure S1: XPS C1s of (a) MFC-700-0.1, (b) MFC-700-0.2, and (c) MFC-750-0.2; Figure S2. Semi-logarithmic N_2_ sorption isotherms of the samples prepared at different conditions. Table S1: N-species contribution in total N obtained from fitting of the N 1s XPS spectra.

## Sample Availability

Samples of the compounds are not available from the authors.
