# Supplementary material for: One-Pot Synthesis of Melamine Formaldehyde Resin-Derived N-Doped Porous Carbon for CO2 Capture Application
Source: Molecules. 2023 Feb 13;28(4):1772. doi: 10.3390/molecules28041772 (PMC9958949; doi:10.3390/molecules28041772)
Supplement: Supplementary file 1 [file molecules-28-01772-s001.zip › molecules-2208370-supplementary.pdf]

One-Pot Synthesis of Melamine Formaldehyde Resin- Derived N-Doped Porous Carbon for CO2 Capture Application

(Supplementary materials)

Qiyun Yu 1, Jiali Bai 1, Jiamei Huang 1, Muslum Demir 2,3,

Ahmed A. Farghaly 4,5, Parya Aghamohammadi 2, Xin Hu 1,* and Linlin Wang 6,*

1 Key Laboratory of the Ministry of Education for Advanced Catalysis Materials, Zhejiang Normal University, Jinhua 321004, China

2 Department of Chemical Engineering, Osmaniye Korkut Ata University, Osmaniye 80000, Turkey

3 TUBITAK Marmara Research Center, Material Institute, Gebze 41470, Turkey

4 Chemical Sciences and Engineering Division, Argonne National Laboratory, Lemont, IL 60439, USA

5 Chemistry Department, Faculty of Science, Assiut University, Assiut 71516, Egypt

6 Key Laboratory of Urban Rail Transit Intelligent Operation and Maintenance Technology and Equipment of Zhejiang Province, College of Engineering, Zhejiang Normal University, Jinhua 321004, China

* Correspondence: huxin@zjnu.cn (X.H.); wanglinlin@zjnu.cn (L.W.); Tel.: 86-151-0579-0257 (X.H.)

IAST CO_2/N_2 selectivity

To calculate the IAST CO_2/N_2 selectivity, the CO_2 adsorption isotherm was fitted with a Langmuir–Freundlich equation and N_2 isotherm was fitted with a linear equation, respectively.

The adsorption selectivity of carbon dioxide over nitrogen was calculated according to the following equation:

$$S = \frac{V_1/V_2}{P_1/P_2}$$

where V_1 and V_2 are the adsorbed amount of carbon dioxide at 0.1 bar and nitrogen at 0.9 bar, respectively, which can be derived from the fitted equation; P_1 and P_2 are the equilibrium partial pressure of carbon dioxide (0.1 bar) and nitrogen (0.9 bar) in the bulk gas phase, respectively.

CO_2 heat of adsorption

CO_2 heat of adsorption was calculated using a variant of the Clausius-Clapeyron equation taking both the 273K and 298K CO_2 adsorption data.

$$\ln\left(\frac{P_1}{P_2}\right) = Q_{st} * \frac{T_2 - T_1}{R * T_1 * T_2}$$

Where P_n : Pressure for isotherm n

T_n: Temperature for the isotherm n

R: Gas constant

Pressure as the function of the adsorbed amount was determined by using Langmuir–Freundlich equation. This Langmuir–Freundlich equation gives an accurate fit over the pressure up to 1 bar and with the goodness of fit (R^2) above 0.99. The corresponding P_1 and P_2 at a certain CO_2 adsorbed amount of both temperatures can

be obtained by the simulated Langmuir–Freundlich equation. Then input these numbers into the above equation, the corresponding CO_2 heat of adsorption was calculated.

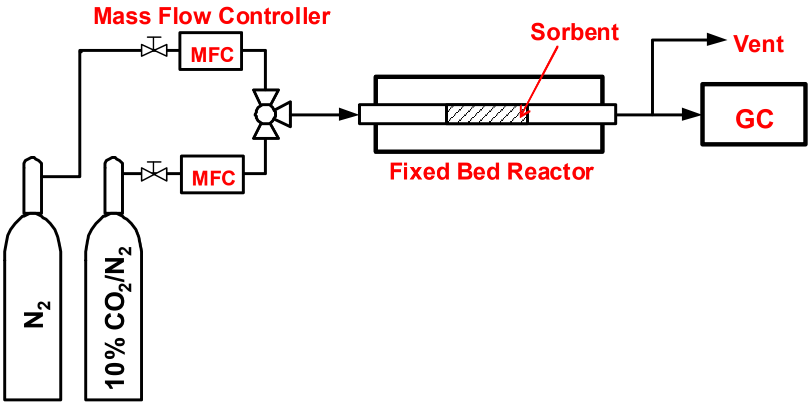

Scheme S1. Schematic of the fixed-bed reactor system.

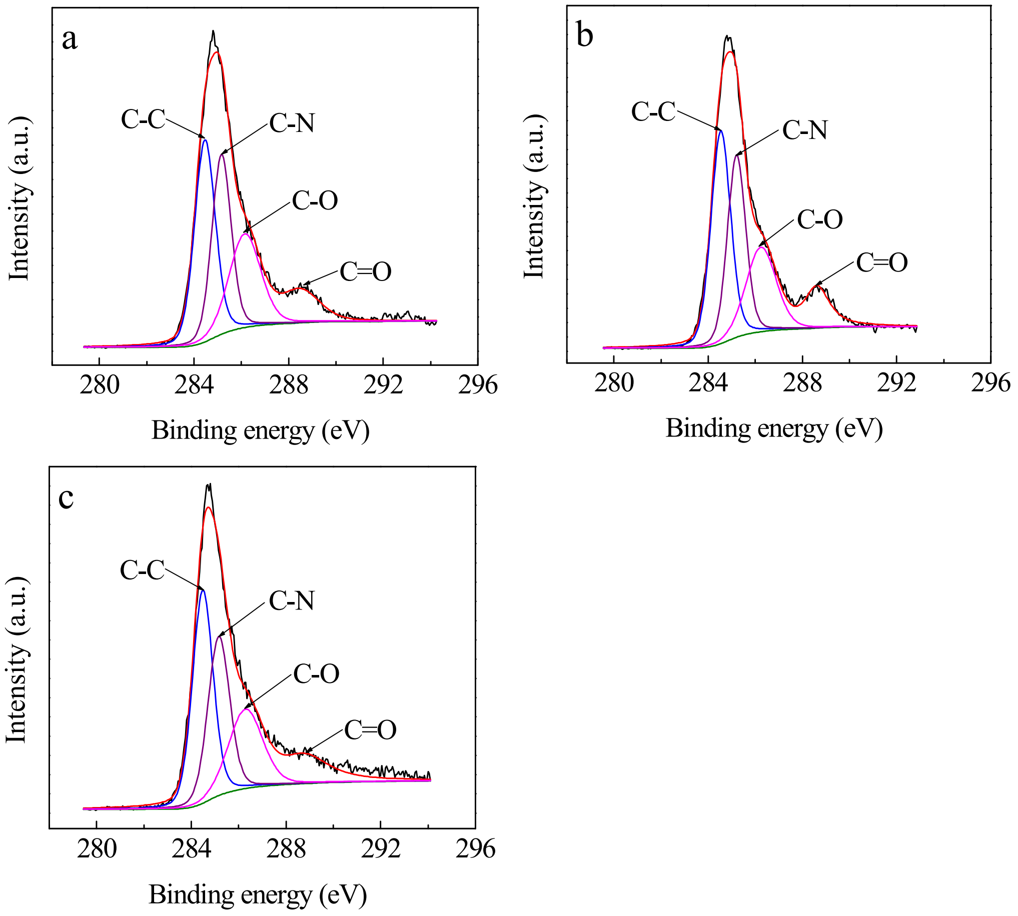

Figure S1. XPS C1s of (a) MFC-700-0.1, (b) MFC-700-0.2, and (c) MFC-750-0.2

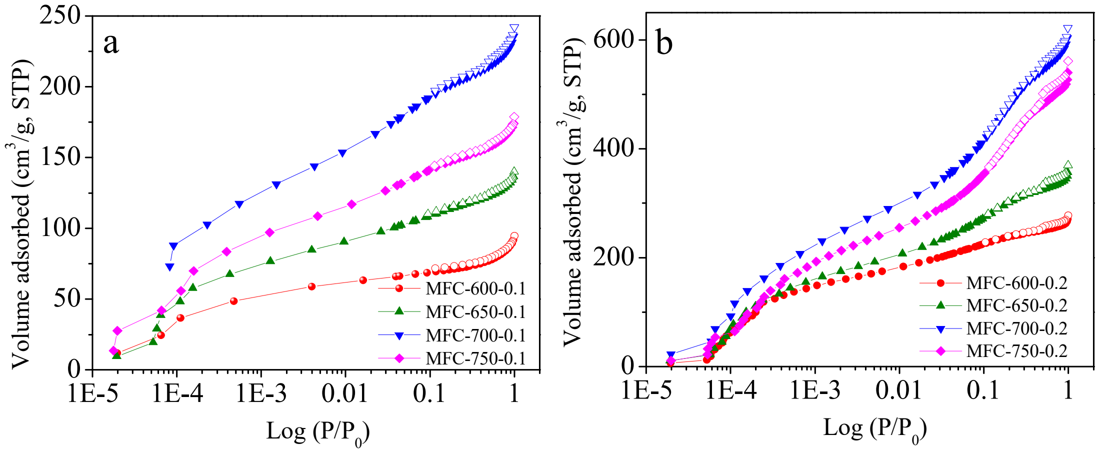

Figure S2. Semi-logarithmic N_2 sorption isotherms of the samples prepared at different conditions. Filled and empty symbols represent adsorption and desorption branches, respectively.

Table S1. N-species contributions in total N obtained from fitting of the N 1s XPS spectra

Sample	N-5 (at.%)	N-6 (at.%)	Graphitic N (at.%)
MFC-700-0.1	53.75	40.03	6.22
MFC-700-0.2	46.51	40.67	12.82
MFC-750-0.2	41.11	43.19	15.6
